# Varicocele Repair Improves Testicular Histology in Men with Nonobstructive Azoospermia

**DOI:** 10.1155/2015/709452

**Published:** 2015-10-27

**Authors:** Murat Ustuner, Hasan Yilmaz, Ufuk Yavuz, Seyfettin Ciftci, Ali Saribacak, Bahri Serkan Aynur, Hikmet Yasar, Mustafa Melih Culha

**Affiliations:** ^1^Department of Urology, School of Medicine, University of Kocaeli, 41380 Kocaeli, Turkey; ^2^Department of Urology, VM Medical Park Hospital, Kocaeli, Turkey; ^3^Department of Urology, Darica Farabi State Hospital, Kocaeli, Turkey

## Abstract

*Objective*. To determine the histopathological differences after varicocele repair in testicular tissue in males with nonobstructive azoospermia. *Methods*. Between 2009 and 2014, 45 men with complete azoospermia and palpable varicocele, presenting with primary infertility of at least 1 year, undergoing varicocele repair at our institution were selected for the study. A standard systematic testicular 6-core Tru-Cut biopsy was performed during varicocele repair. Other biopsies were obtained from each testicle of all patients at the time of microscopic sperm extraction procedure. *Results*. Nineteen patients were selected for the study. Testicular biopsy specimens were classified as Sertoli cell only on preoperative histopathological analysis in 14 patients. After varicocele repair, focal spermatogenesis (*n* = 3) and late maturation arrest (*n* = 2) were found in these patients. Average Johnsen score was significantly increased after varicocelectomy (*P* = 0.003). Motile sperm was found in one patient on postoperative semen analyses and in 10 more patients in the microscopic sperm extraction procedure. Preoperative high serum follicle stimulating hormone level and venous reflux were significantly and negatively correlated with the increase in average Johnsen score (*P* < 0.05). *Conclusions*. Our findings suggest significant improvement in testicular histology after varicocele repair.

## 1. Introduction

There is no standard treatment for male infertility. Varicocele is the most common cause of male infertility and is generally correctable or at least improvable by various surgical and radiological techniques. It has been estimated that 5–10% of infertile males with azoospermia had a clinical diagnosis of varicocele [1, 2]. Urology guidelines recommend varicocele repair (VR) in infertile patients with semen abnormalities and palpable varicocele [3]. The benefits of VR for sperm concentration, motility, and morphology are well established in oligozoospermic males [4], but the efficacy of VR on testicular histology changes in cases of nonobstructive azoospermia (NOA) has not been examined yet.

Fathering a child for males with NOA is directly associated with obtaining spermatozoa by microscopic testicular sperm extraction (micro-TESE) and the success of intracytoplasmic sperm injection (ICSI). ICSI is the only way for males with NOA to have children. However, ICSI results in successful pregnancy for only a small percentage of males with NOA. Pregnancies and live births are eventually achieved in 30–50% of couples in which the male has NOA, when spermatozoa have been found on testicular biopsy [3]. Sperm recovery rates for ICSI treatment differ between 30 and 70% in various studies [5]. Consequently, auxiliary treatments are required to improve the recovery of testicular tissue, quality of spermatozoa, and probability of obtaining spermatozoa.

Tulloch first applied VR in one NOA patient with bilateral varicocele in 1952 and obtained spontaneous pregnancy [6]. After that, also other studies were published regarding VR in NO patients [1, 2, 7]. Subsequently, a number of studies were performed to determine the pathophysiology of varicocele and the role of VR in recovery of fertility. We examined the histopathological differences associated with VR in testicular tissue. The parameters predictive of postoperative improvement in men with NOA were also determined in this study.

## 2. Materials and Methods

The study was carried out according to the Declaration of Helsinki and was approved by our institutional ethics committee with the number of IAEK 7/3-2009/54. Between 2009 and 2014, men with pellet (−) azoospermia and palpable unilateral or bilateral varicocele, presenting with primary infertility for at least 1 year, were selected for this study. All subjects underwent a standard basic diagnostic infertility evaluation. A detailed medical history was obtained, and a physical examination for complete infertility evaluation was performed. Varicoceles were identified on scrotal examination and classified as described previously [3]. Scrotal ultrasonography with real-time color Doppler imaging was used to confirm the presence of varicocele. At least three preoperative semen samples were obtained from all patients by masturbation after 4-5 days of abstinence. All analyses were performed in the same andrology laboratory (Kocaeli University Infertility Center).

All patients had the normal 46XY karyotype and did not have any Y-microdeletions. Patients with obstructive azoospermia, retrograde ejaculation, history of systemic disease and/or surgery that may affect testicular histology, and low serum testosterone levels were excluded. Patients' age, serum follicle stimulating hormone (FSH) values before surgery, grade and laterality of varicocele, time interval between varicocelectomy and micro-TESE, maximum diameter of varicose veins, and presence of retrograde flow (venous reflux) were obtained.

Informed consent was obtained from all participants. A total of 45 patients underwent microsurgical VR with subinguinal approach. After the VR a 5-mm midline dermal and subdermal scrotal incision was done, and a standard systematic testicular 6-core Tru-Cut biopsy (from each polar and midline on the right and left testicle) with a 20-mm 18-gauge needle was performed. Biopsies were laid separately on absorbent paper and placed into Eppendorf tubes filled with Bouin's solution and transferred to the pathology laboratory. Tissues were embedded in paraffin blocks after processing and cut into sections 5 *μ*m thick, which were deparaffinized and stained with hematoxylin and eosin. All pathological analyses were performed by an expert uropathologist (KY) (preoperative histopathology). Any tissue obtained from the biopsy was not cryopreserved.

More than one semen sample was obtained from each patient beginning three mounts after the surgery. After complete gynecological evaluation of their partners, all patients presented to Kocaeli University Assisted Reproduction Treatment Center. Eighteen patients were not engaged in assisted reproductive treatments for financial reasons. During the evaluation of some biopsies of six patients, uropathologist could not find any testicular tissue, probably due to some technique issues. So these six and two patients with normal spermatogenesis in their preoperative biopsy were excluded. Micro-TESE and ICSI were performed for the remaining 19 subjects.

Micro-TESE procedure: Micro-TESE was performed under local anaesthesia by removing testicular tissue through a longitudinal incision of tunica albuginea. The testicular pulp was surged under operative microscope for dilated and enlarged tubules which are more likely to contain germ cells. Testicular tissues were obtained from different parts and levels.

Regardless of the success of the micro-TESE procedure, all extracted testicular tissues were sent to pathology laboratory for histopathological evaluation. Samples were put in tubes filled with Bouin's solution and were transferred to the pathology laboratory for histological evaluation (postoperative histopathology).

Histology was analyzed by scoring the seminiferous tubules at 400x magnification using the Johnsen score (JS) (10) according to the presence of germinal cells. All histopathological evaluation (Tru-Cut biopsies and micro-TESE material) was performed using JS. Each tubular section was given a score from 10 to 1 according to the presence or absence of the main cell types arranged in the order of maturity: scores 10, 9, or 8, presence of spermatozoa; 7 or 6, spermatids (and no further); 5 or 4, spermatocytes (and no further); 3, only spermatogonia; 2, only Sertoli cells; and 1, no cells. The germinal epithelium of at least 10, maximum 20, tubules was assessed for each testis, and the average Johnsen score was calculated for each patient. Testicular biopsy specimens were classified according to the histopathological criteria as follows: normal spermatogenesis (NS), hypospermatogenesis (HS), late maturation arrest (LMA), early maturation arrest (EMA), Sertoli cells only with focal spermatogenesis (SCO-FS), Sertoli cell only (SCO), and hyalinization of tubules (HT).

### 2.1. Statistical Analysis

Preoperative high (above the reference value) FSH level, presence of venous reflux and bilateral varicocele, and presence of increased average JS in postoperative histopathology were modeled as dichotomous variables (yes/no). When bilateral varicocele was determined, the higher grade was used in the analysis. All data were analyzed using SPSS. The Wilcoxon matched-pair signed ranks test was used for nonparametric analyses. Univariate logistic regression (LR) analyses were performed and the presence of increased average JS was used as a dependent variable. In all analyses, *P* < 0.05 was taken to indicate statistical significance.

## 3. Results

Only patients having both preoperative and postoperative histopathological evaluation were included in analyses. Thus, we analyzed data on 19 patients. All patients had a diagnosis of the pellet (−) NOA and had palpable varicocele. The preoperative patient characteristics are shown in Table 1. Serum FSH level was high in 12 patients. Varicose veins of maximum diameter >3 mm were found in all patients.

**Figure 1:**
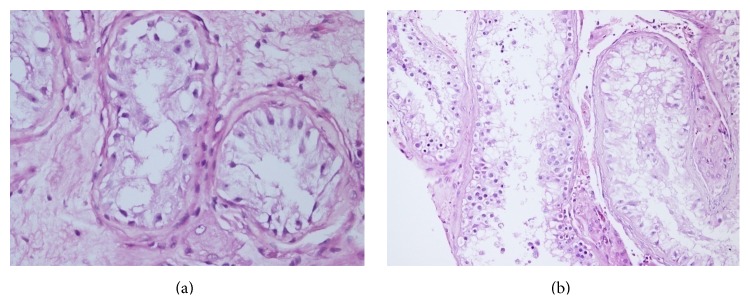
(a) Testicular histopathology indicating SCO. (b) Testicular histopathology showing SCO pattern with focal spermatogenesis in the same patient after VR.

The median of average JS before and after VR was 2.00 (interquartile range (IQR) 2.00–2.40) and 3.30 (IQR 2.00–5.10), respectively. The average JS was significantly increased after VR (*P* = 0.003). The percentages of histopathological findings according to preoperative and postoperative testicular biopsies are shown in Table 2. Preoperative SCO was defined in 14 patients, focal spermatogenesis in two, hypospermatogenesis in two, and EMA in one. After VR, focal spermatogenesis was determined in three and LMA in two patients with SCO (Figure 1). Motile sperm were found in 1 patient on postoperative semen analyses and in a further 8 patients by the micro-TESE procedure. Average JS increased in 10 subjects and was unchanged in 9 (patients with SCO).

Univariate logistic regression analyses were performed to assess the factors correlated with the increased average JS (Table 3). Preoperative high serum FSH level and venous reflux were significantly correlated with failure to increase average JS (*P* < 0.05).

## 4. Discussion

The role of varicocelectomy in azoospermic patients was first studied in 1976 by Mehan [8]. He applied spermatic vein ligation to 10 NOA patients (2 of whom had varicocele) and confirmed two spontaneous pregnancies. Many subsequent studies have evaluated this issue [1, 2, 7, 9–12]. Weedin et al. performed a meta-analysis to evaluate studies of VR in patients with NOA performed during the previous 20 years [13]. They analyzed 11 publications and a total of 233 patients. Motile sperm were found on postoperative semen analyses in 91 of 233 (39.1%) patients, resulting in 14 (6%) spontaneous pregnancies [13]. Inci et al. and Haydardedeoglu et al. retrospectively evaluated NOA patients with or without VR in two separate studies. They compared the presence or absence of motile sperm in the TESE procedure and found significantly higher rates of motile sperm in patients with varicocelectomy [14, 15].

Our five patients with a preoperative diagnosis of SCO had focal spermatogenesis (FS) and LMA on histopathological evaluation of micro-TESE specimens after VR. There have been several reports regarding motile sperm obtained in SCO patients. Pasqualotto et al. [16] found motile sperm in 4 of 10 SCO patients and Lee et al. [17] found motile sperm in 1 of 10 SCO patients, in their study. However, Kim et al. reported that three patients with the SCO pattern and three with EMA showed no improvement after varicocelectomy, while 5 of 10 (50%) with LMA and 10 of 18 (56%) with HS did show improvement. Similarly, Kadioğlu et al. and Çakan and Altuğ obtained no motile sperm after VR in SCO patients [7, 18]. Weedin et al. reported motile sperm in 5 of a total of 44 SCO patients, whereas motile sperm were detected in 30 of 55 HS and 24 of 57 maturation arrest patients in their meta-analysis [13].

There might be two possible explanations for detection of motile sperm in SCO patients. First, nonproductive testicular tissue would recover after VR and inactive germ cell precursors would be activated; the observation of focal spermatogenesis after VR in three preoperative complete SCO patients might support this hypothesis. Second, existing spermatogenesis may not have been detected during testicular biopsy and the improvement in histopathology might be a reason of more extensive tissue analysis at the time of micro-TESE procedure. It is well established that spermatogenesis can vary within a compromised testicle. Therefore, testicular biopsies may not always be representative of the most advanced histological pattern within the testis. However, we performed systematic testis biopsy in both testes with six samples in our prospective study design instead of obtaining only one biopsy from any testis. This was expected to lead to more representative findings than conventional testis biopsies. Nevertheless, although less likely, preoperative biopsy may be unable to detect existing spermatogenesis in the testicular tissue.

To our knowledge, this is the first report to compare the histopathological findings before and after VR. Although two thirds of our patients had preoperative SCO, we found a significant increase in the average JS. None of the patients showed a decrease in average JS. In conclusion, our findings represent clear evidence that VR positively affected the testicular histology. Histological changes were determined only in SCO group in our study. However, most of the patients in our study had this histology. We expect that larger study groups with different histologies might have represent improvement in all patients.

We evaluated the predictive factors according to histopathological improvement, unlike other studies, and our results were different from those reported previously. In the present study, we found that a preoperative high serum FSH level and the presence of venous reflux in color Doppler imaging negatively affected the testicular histopathological improvement. Similarly, Kadioğlu et al. showed that patients with normal FSH levels had higher rates of improvement in semen analyses [7]. However, several studies reported that a high FSH level was not associated with obtaining motile sperm after VR in NOA patients [1, 2, 18–20]. No previous study has evaluated whether the diameter of varicose veins and the presence of venous reflux affect the success of VR in NOA patients. However, in patients with varicocele and oligoasthenospermia, testicular vein diameter > 2.5 mm and the presence of venous reflux in preoperative color Doppler ultrasound were associated with significant improvements in sperm parameters after VR [21, 22].

This study has a number of limitations. There is not a control group in our study. But it is not easy to generate a real control group because of limited number of patients and much of the patients in our clinic with palpable varicocele and NOA did not accept to undergone testicular biopsy and VR before micro-TESE procedure. However some of these patients who had negative ICSI procedure were convinced of VR after the unsuccessful treatment. These patients might be used as a control group in another study and testicular histology can be compared before and after VR in the future. On the other hand, it is well established that spermatogenesis can vary within a compromised testicle. Although we performed systematic testicular biopsy, it would not be representative of the overall testicular tissue. In addition, the time interval between VR and micro-TESE was relatively long.

## 5. Conclusion

To our knowledge, this is the first report to compare the histopathological findings before and after VR in males with NOA. Our findings suggested significant improvement in testicular histology regarding average JS after VR. In addition, preoperative high FSH levels and the presence of venous reflux were negatively correlated with this improvement.

## Figures and Tables

**Table 1:** Patient characteristics.

Age (year), [median (IQR^*∗*^)]	31.00 (29.00–35.00)

Serum FSH^*∗∗*^ level (mIU/mL), [median (IQR)]	26.50 (9.24–41.50)

The time interval between varicocelectomy and micro-TESE (month), [median (IQR)]	12.00 (8.00–20.00)

Maximum diameter of veins (mm), [median (IQR)]	3.20 (2.80–3.50)

Presence of venous reflux, % (*n*)	47.4 (9)

Varicocele grade, % (*n*)	
Grade I	21.1 (4)
Grade II	52.6 (10)
Grade III	26.3 (5)

Varicocele laterality, % (*n*)	
Unilateral	52.6 (10)
Bilateral	47.4 (9)

^*∗*^IQR: interquartile range.

^*∗∗*^FSH: follicle stimulating hormone.

**Table 2:** The percentages of histopathological findings according to preoperative and postoperative testicular biopsies.

Histopathology	Preoperative	Postoperative
	% (*n*)	% (*n*)
HS	10.5 (2)	10.5 (2)
SCO-FS	10.5 (2)	26.3 (5)
LMA	0	10.5 (2)
EMA	5.3 (1)	5.3 (1)
SCO	73.7 (14)	47.4 (9)

HS: hypospermatogenesis, LMA: late maturation arrest, EMA: early maturation arrest, SCO-FS: Sertoli cells only with focal spermatogenesis, SCO: Sertoli cell only.

**Table 3:** The predictive factors for improvement of average JSs in univariate logistic regression analyses.

	OR (CI)	*p*
Age	0.93 (0.78–1.12)	0.466
Pre-op high serum FSH^*∗*^ level	0.08 (0.07–0.95)	**0.045**
The interval between surgery and micro-TESE	1.16 (0.99–1.35)	0.054
Varicocele grade		
Grade I	2.00 (0.11–35.80)	0.638
Grade II	0.44 (0.05–3.97)	0.468
Grade III	1.50 (0.18–11.92)	0.702
Presence of bilateral varicocele	0.21 (0.03–1.48)	0.119
Maximum diameter of varicose vein	0.30 (0.02–3.13)	0.317
Presence of venous reflux	0.07 (0.01–0.64)	**0.019**

^*∗*^FSH: follicle stimulating hormone.
